# Supplementary material for: Differences in the genetic control of early egg development and reproduction between C. elegans and its parthenogenetic relative D. coronatus
Source: EvoDevo. 2017 Oct 18;8:16. doi: 10.1186/s13227-017-0081-y (PMC5648466; doi:10.1186/s13227-017-0081-y)
Supplement: Supplementary file 1 — Additional file 1: Table S1. RNA-Seq statistics. Table S2: dN/dS values. [file 13227_2017_81_MOESM1_ESM.docx]

**Table S1**: Retrieved paired-end reads from RNA sequencing of four independent D. coronatus samples

| **Library^a^** | **paired-end reads** | **read length [bp]** | **discarded paired-end reads^b^** | **successfully mapped reads^c^** | **assembled and mapped transcripts** | **Illumina platform** |
| --- | --- | --- | --- | --- | --- | --- |
| #1 | 6,979,616 | 75 | 3,402,448 | 173,936 (2.5%) | 1,059 | MiSeq |
| #2 | 8,898,677 | 75 | 7,947,398 | 673,291 (7.6%) | 3,151 | MiSeq |
| #3 | 31,199,716 | 100 | 1,111,574 | 1,779,470 (6.0%) | 4,550 | HiSeq |
| #4 | 28,416,086 | 100 | 1,341,994 | 1,349,244 (5.0%) | 5,384 | HiSeq |

^a^, each sample >100 early embryos (1- to 8-cell stages); ^b^, paired-end reads with low sequencing quality for the 5'- and/or 3'-ends were removed; c, successfully mapped raw reads to the *D. coronatus* EST library and transcriptome.

**Table S2:** Ratio of non-synonymous to synonymous base exchanges in 11 conserved single copy genes of D. coronatus.

**Fosmid ID^a^ *C.e.* gene dN/dS**

**name**

C09G12.9 *tsg-101* 0.096

F08D12.1 *srpa-72* 0.144

F21H11.2 *sax-2* 0.842

F32E10.1 *nol-10* 0.129

F39C12.1 F39C12.1 0.388

F41E6.9 *vps-60* 0.022

F44A6.1 *nucb-1* 0.093

F48F5.5 *fce-2* 0.337

F53G2.6 *tsr-1* 0.158

Y47D3A.28 *mcm-10* 0.284

Y65B4A.1 Y65B4A.1 0.277

median 0.158

^a^, orthologs taken from Mitreva et al., 2011
