# Supplementary figures and images for: Differences in the genetic control of early egg development and reproduction between C. elegans and its parthenogenetic relative D. coronatus
Source: EvoDevo. 2017 Oct 18;8:16. doi: 10.1186/s13227-017-0081-y (PMC5648466; doi:10.1186/s13227-017-0081-y)

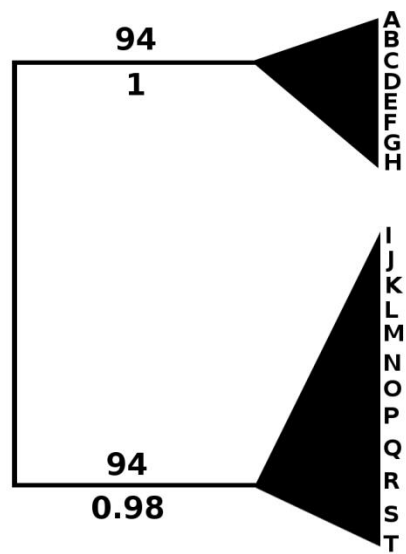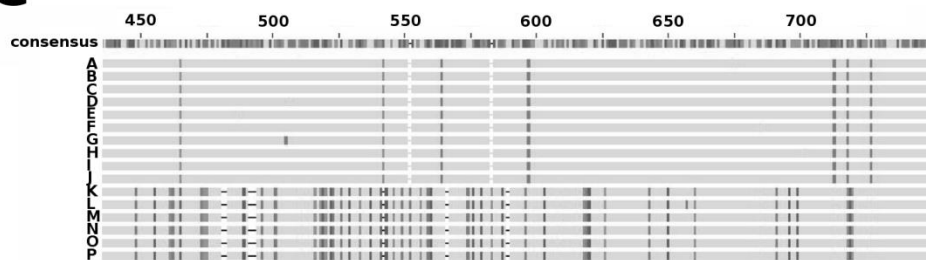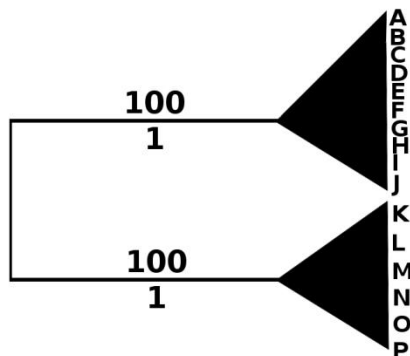

Supplement: Supplementary file 2 — Additional file 2: Fig. S2. a, c, Sequence comparison of the small (SSU) and large subunit (LSU) rDNA genes of D. coronatus. b, Collapsed Maximum Likelihood (ML) tree representing clustering of sequenced clones for the SSU rDNA gene. (d) Collapsed ML tree representing clustering of sequenced clones for the LSU rDNA gene. Bootstrap values are shown above and posterior probability values beneath branches. [file 13227_2017_81_MOESM2_ESM.pdf]

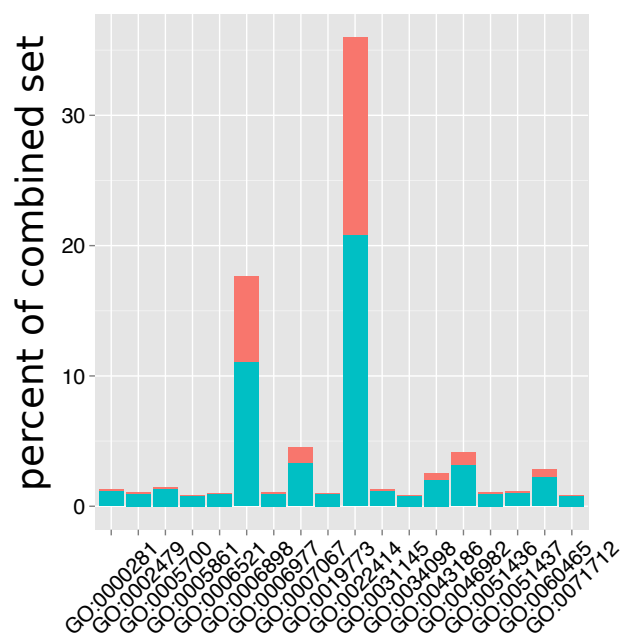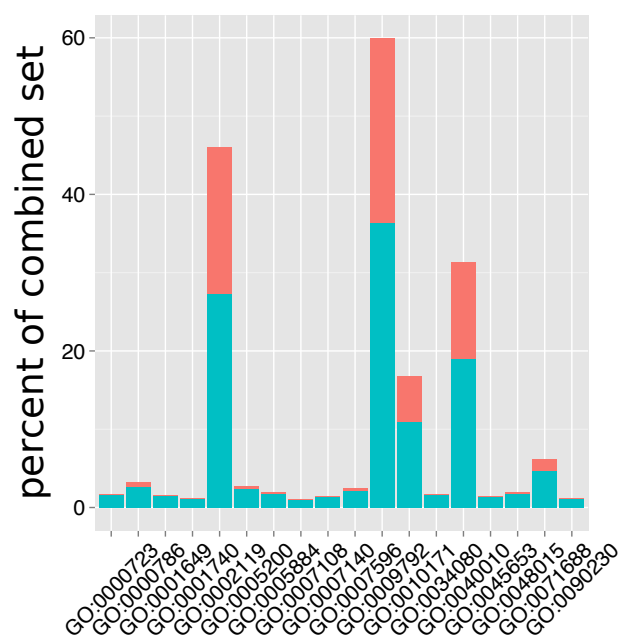

■ Test set: 1-8 cell stage *D. coronatus* proteome

Supplement: Supplementary file 3 — Additional file 3: Fig. S1. GO terms enriched in the D. coronatus 1–8 cell transcriptomic proteome in comparison with the complete D. coronatus proteome. Associated functional descriptions and test statistics are given in tabular format in Additional file 4. [file 13227_2017_81_MOESM3_ESM.pdf]

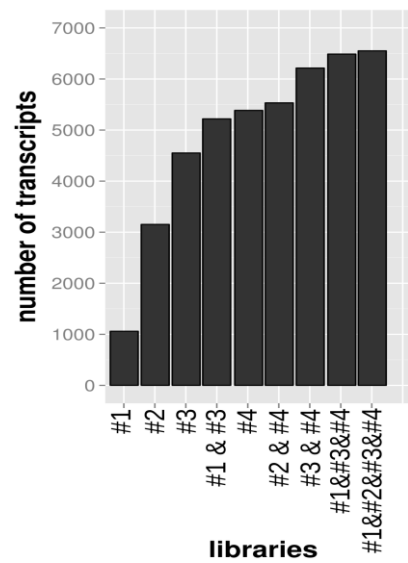

Supplement: Supplementary file 5 — Additional file 5: Fig. S3. Binning of different combinations of replicates. Combining all four replicates the numbers of expressed sequences appear to saturate at about 6500 transcripts (see Table 2). [file 13227_2017_81_MOESM5_ESM.pdf]

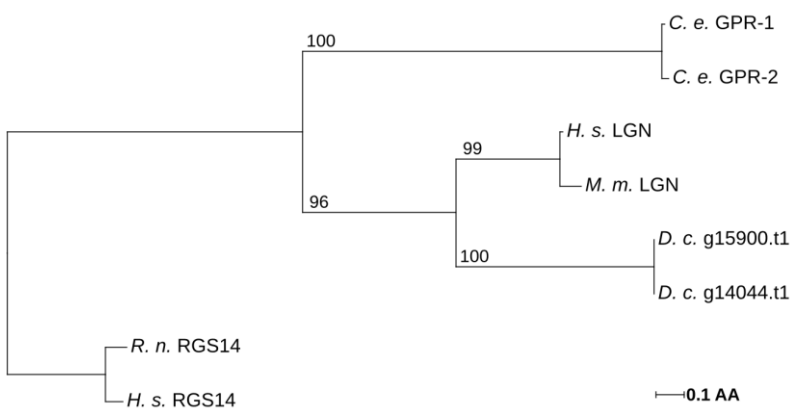

Supplement: Supplementary file 6 — Additional file 6: Fig. S4. Phylogenetic tree representing GoLoco (Pfam ID PF02188) domain proteins of D. coronatus (D. c.), C. elegans (C. e.), human (H. s.), rat (R. n.) and mouse (M. m.). [file 13227_2017_81_MOESM6_ESM.pdf]
